# Digital PCR Improves Mutation Analysis in Pancreas Fine Needle Aspiration Biopsy Specimens

**DOI:** 10.1371/journal.pone.0170897

**Published:** 2017-01-26

**Authors:** Shonan Sho, Colin M. Court, Stephen Kim, David R. Braxton, Shuang Hou, V. Raman Muthusamy, Rabindra R. Watson, Alireza Sedarat, Hsian-Rong Tseng, James S. Tomlinson

**Affiliations:** 1 Department of Surgery, University of California Los Angeles, Los Angeles, California, United States of America; 2 Department of Surgery, Greater Los Angeles Veteran’s Affairs Administration, Los Angeles, California, United States of America; 3 Department of Molecular and Medical Pharmacology, University of California Los Angeles, Los Angeles, California, United States of America; 4 UCLA Center for Pancreatic Diseases, University of California Los Angeles, Los Angeles, California, United States of America; 5 Division of Digestive Diseases, David Geffen School of Medicine at the University of California Los Angeles, Los Angeles, California, United States of America; 6 Department of Pathology, University of California Los Angeles, Los Angeles, California, United States of America; Universita degli Studi di Verona, ITALY

## Abstract

Applications of precision oncology strategies rely on accurate tumor genotyping from clinically available specimens. Fine needle aspirations (FNA) are frequently obtained in cancer management and often represent the only source of tumor tissues for patients with metastatic or locally advanced diseases. However, FNAs obtained from pancreas ductal adenocarcinoma (PDAC) are often limited in cellularity and/or tumor cell purity, precluding accurate tumor genotyping in many cases. Digital PCR (dPCR) is a technology with exceptional sensitivity and low DNA template requirement, characteristics that are necessary for analyzing PDAC FNA samples. In the current study, we sought to evaluate dPCR as a mutation analysis tool for pancreas FNA specimens. To this end, we analyzed alterations in the *KRAS* gene in pancreas FNAs using dPCR. The sensitivity of dPCR mutation analysis was first determined using serial dilution cell spiking studies. Single-cell laser-microdissection (LMD) was then utilized to identify the minimal number of tumor cells needed for mutation detection. Lastly, dPCR mutation analysis was performed on 44 pancreas FNAs (34 formalin-fixed paraffin-embedded (FFPE) and 10 fresh (non-fixed)), including samples highly limited in cellularity (100 cells) and tumor cell purity (1%). We found dPCR to detect mutations with allele frequencies as low as 0.17%. Additionally, a single tumor cell could be detected within an abundance of normal cells. Using clinical FNA samples, dPCR mutation analysis was successful in all preoperative FNA biopsies tested, and its accuracy was confirmed via comparison with resected tumor specimens. Moreover, dPCR revealed additional *KRAS* mutations representing minor subclones within a tumor that were not detected by the current clinical gold standard method of Sanger sequencing. In conclusion, dPCR performs sensitive and accurate mutation analysis in pancreas FNAs, detecting not only the dominant mutation subtype, but also the additional rare mutation subtypes representing tumor heterogeneity.

## Introduction

Cancer is a disease of genetic aberrations.[1] Tumors occurring in the same anatomical location or having similar histologic features have distinct clinical behaviors, responses to therapy and outcomes depending on their genetic profiles. Thus, there is now a well recognized need to move beyond the traditional treatment guidelines based on tumor origin and histopathologic characteristics, focusing instead on a precision oncology approach: genetically identifying the patient that is likely to benefit from a given agent or treatment.[2] To this end, the NCI Molecular Analysis for Therapy Choice (NCI-MATCH) trial is currently underway to link cancer genomic abnormalities to specific molecularly targeted cancer drugs. Furthermore, the precision oncology approach is becoming a part of the common clinical practice for various cancer types, including breast, colorectal and non-small cell lung carcinoma.

The success of the NCI-MATCH initiative, and of the precision oncology strategy in general, hinges on the ability to obtain accurate genetic information from tumor specimens. Assignment of an appropriate therapy based on tumor genetic aberrations cannot occur without an effective strategy for tumor genotyping. In clinical practice, tumor tissues are obtained through either surgical excision or image guided biopsy, most commonly a fine needle aspiration (FNA). For a significant subset of patients, surgery is precluded due to metastatic or locally advanced disease, making FNA biopsy the only potential source of tumor tissue. This can present a major challenge to the implementation of a precision oncology strategy, as cellular and DNA yields from these FNA biopsy samples are sometimes inadequate for routine molecular analysis.[3, 4] While systemic therapies are most useful in patients with metastatic or locally advanced diseases, it is in these patients that the tumor tissues available for guiding precision oncology strategies are limited.

Accurate tumor genotyping is especially challenging in patients with pancreas ductal adenocarcinoma (PDAC) due to the large non-cancer (stromal) component that is characteristic of PDAC primary tumors.[5] When endoscopic ultrasound guided-FNA (EUS-FNA) is performed for these tumors, the resulting biopsy specimens often contain mostly stromal cells and few actual tumor cells.[6] Furthermore, the large excess of wild-type (WT) DNA from abundant normal stromal cells can overshadow the mutant DNA from rare PDAC tumor cells, making the mutant allele fraction too small to be detected. For example, Sanger sequencing, the gold standard for clinical sequencing, requires approximately 150-200ng of starting DNA and has a mutant allele fraction detection limit of approximately 10%.[7, 8] Thus, many EUS-FNAs from PDAC masses result in samples that cannot be accurately genotyped by this conventional technique.[9]

Two major hurdles to the routine clinical use of pancreas EUS-FNA specimens in molecular analysis exist: insufficient DNA content and low tumor cell purity. While some EUS-FNA samples prove adequate in both of these aspects, many aspirates are insufficient in one or both of them. A newly developed PCR platform, digital PCR, has the potential to overcome these problems.[10] Digital PCR (dPCR) is a technology based on the principle of compartmentalization and parallel PCR reactions. By distributing a DNA sample into many compartments such that only a single copy of the target DNA is present within each compartment, a rare mutant DNA can be amplified and detected individually without being lost in the large excess of WT DNA. Furthermore, by counting each compartment containing the target mutant DNA, absolute quantification and relative frequencies of mutant to WT DNA alleles can be determined. This technique can perform highly sensitive mutation detection with minimal template DNA input, enabling accurate and sensitive genotyping using tumor biopsy samples that are limited in both quantity and tumor cell purity. [10–12]

Given these strengths, dPCR holds the potential to become an effective tool for mutation analysis in pancreas EUS-FNA biopsy specimens. To evaluate this application, we analyzed alterations in the *Kirsten rat sarcoma viral oncogene homolog* (*KRAS*) gene in pancreas FNA specimens using dPCR. *KRAS* mutations are present in over 95% of PDAC tumors.[13] Additionally, the *KRAS* mutation is considered a driver mutation as it occurs early in carcinogenesis and thus should be present in all cancer cells. These factors make the *KRAS* gene in PDAC an ideal target to analyze, as the ubiquity of *KRAS* mutation allows us to readily assess the mutation detection capabilities of dPCR. Furthermore, approximately 10–20% of PDAC patients are known to harbor multiple *KRAS* mutation subtypes within a single PDAC mass, representing the presence of distinct tumor subclones and intratumoral heterogeneity.[9, 14] Thus, testing for the *KRAS* gene also reveals whether dPCR could accurately portray the intratumoral heterogeneity of a PDAC mass using only FNA biopsy specimens. Lastly, recent findings suggest that an accurate determination of *KRAS* mutation subtypes in and of itself holds potential importance in PDAC management. Multiple reports have implicated specific *KRAS* mutation subtypes in prediction of prognosis and therapy responses. [15–18] Additionally, *KRAS* subtype-specific inhibitors are being developed.[19] Thus, *KRAS* mutation subtyping in PDAC patients has the potential to guide precision oncology strategies in the near future, similar to the way *KRAS* mutations have emerged as predictors of therapy response in non-small cell lung carcinoma and colon cancer patients.

In the current study, we evaluate the use of dPCR for mutation analysis in pancreas EUS-FNA specimens, focusing specifically on the *KRAS* gene for the above reasons. To this end, we first evaluated the mutation detection sensitivity and minimal cellular input requirement of dPCR. Next, we used dPCR to perform *KRAS* mutation analysis in both formalin-fixed paraffin embedded (FFPE) and non-fixed, fresh EUS-FNA pancreas biopsy specimens. Using DNA extracted from formalin-fixed tissues introduces an additional challenge of DNA degradation. However, the ability to use FFPE EUS-FNA biopsy specimens offers the advantage of avoiding a repeat invasive procedure for the sole purpose of genetic testing. Using fresh EUS-FNA samples, on the other hand, allows for a rapid mutation status analysis immediately following a diagnostic procedure, which can aid physicians in diagnosis and point-of-care decision-making. Lastly, we compared the performance of dPCR against Sanger sequencing, the current gold standard for clinical sequencing, in order to assess whether dPCR could improve the sensitivity and accuracy of *KRAS* mutation detection in EUS-FNA pancreas biopsies.

## Methods and Materials

### Cell lines

Pancreatic cancer cell lines, HPAF-II (CRL-1997) and CFPAC (CRL-1918), T lymphocyte cell line, Jurkat (TIB-152), and breast cancer cell like, MDA-MB-231 (HTB-26), were obtained from American Type Culture Collection (ATCC), and grown using EMEM (HPAF-II), IMDM (CFPAC), RPMI 1640 (Jurkat) and Leibovitz’s L-15 (MDA-MB-231) medium (ATCC) supplemented with 10% fetal bovine serum (ATCC) and 100 U/mL penicillin-streptomycin (ATCC). All cell lines were grown at 37°C with 5% CO_2_ and were routinely passaged at 80% confluence using an iso-osmotic sodium citrate solution for cell release (Thermo). When preparing cells for laser microdissection, cells were released from the culture plates using the same sodium citrate solution. Following a wash with the culture medium, each cell line was diluted to a density of 1000 cells per 100 μL. Approximately 1000 cells (100 μL) were smeared on PEN membrane slides (Leica), air-dried for 10 minutes, and fixed with 100 μL of 100% Ethanol. Cells were then isolated by laser microdissection as outlined below. DNA was extracted using the Qiagen Blood and Cell Culture DNA Mini Kit according to the manufacturer’s protocol. For extraction of DNA from < 100 cells, cell lysis solution from Qiagen RepliG Single Cell kit was used according to the manufacturer’s protocol.

### FFPE pancreas tissue specimens

Archived FFPE blocks containing pancreas tissue specimens were obtained from UCLA Translational Pathology Core Laboratory (TPCL) with the approval from UCLA IRB #13–001646. A total of 63 FFPE blocks were obtained from 34 patients. All 34 patients underwent EUS-FNA biopsy of pancreas lesions and cell blocks were prepared at the time of EUS-FNA procedure according to our institution’s routine clinical protocol. Cytological diagnosis categorized 19 of these cases as “adenocarcinoma,” 10 as “atypical cells” and 5 as “benign.” All patients with cytological diagnosis of either “adenocarcinoma” or “atypical cells” underwent surgical resection of the pancreas. As a result, FFPE blocks from both EUS-FNA pancreas biopsy and pancreas surgical resection were available for the 29 of 34 patients (5 patients with “benign” diagnosis on EUS-FNA biopsy did not receive surgery).

A single 10um section was obtained from each FFPE block, followed by H&E staining performed by UCLA TPCL. Cellularity and tumor location within a 10um section of FFPE EUS-FNA pancreas biopsy specimen was determined by a clinical cytopathologist under high power field. DNA was extracted from a single 10um FFPE section per specimen using GeneRead DNA FFPE extraction kit (Qiagen) according to the manufacturer’s protocol. Extracted DNA was subjected to either Sanger sequencing or digital PCR for KRAS mutation analysis using the methods detailed below.

### Fresh EUS-FNA biopsy specimens

Fresh EUS-FNA biopsy specimens were obtained with the approval from University of California Los Angeles institutional review board, under IRB #13–001646. Written consents were obtained from patients. EUS was performed using a linear echoendoscope (Olympus). Fine needle aspiration was carried out using 25-gauge needles. Aspirated specimens were placed immediately into a 1.5ml microcentrifuge tube in 1ml of normal saline and kept on ice. The specimen was processed within 30 minutes from the EUS-FNA biopsy procedure for DNA extraction using Qiagen Blood and Cell Culture DNA Mini Kit according to the manufacturer’s protocol. The extracted DNA was subjected to both Sanger sequencing and digital PCR, as outlined below.

To confirm the results obtained from dPCR *KRAS* mutation analysis in fresh EUS-FNA biopsy specimens, laser microdissection (LMD) and whole genome amplification (WGA) were used to enhance tumor cell purity and DNA content such that confirmatory. Sanger sequencing could be performed. We obtained FFPE sections from cell blocks prepared at the time of the same EUS-FNA procedures. A single 10um section was made from each block, placed onto PEN membrane slides and H&E stained. LMD was performed to isolate areas with high amount of cancerous cells compared to normal cells in order to enhance tumor purity, as outline below. DNA was then extracted from these microdissected FFPE tissues, and whole genome amplification performed using the PicoPLEX kit (Rubicon) according to the manufacturer’s protocol. Amplified DNA was then purified using QIAquick PCR purification kit (Qiagen) and Sanger sequencing was performed as outlined below. This allowed confirmatory Sanger sequencing in a high tumor purity sample and helped confirm the findings noted in dPCR.

### Laser microdissection

The PALM MicroBeam laser microdissection system (Zeiss) was used for laser microdissection (LMD). Cells were laser dissected and collected into 200 μL opaque tube caps (Zeiss) using the laser pressure catapult function. Cell transfer to the tube cap was confirmed by imaging the cap prior to cap closure using the cap-check function. Zeiss Liquid Cover Glass (Zeiss) was used to improve visualization on H&E stained sections prior to performing LMD.

### *KRAS* PCR and sanger sequencing

PCR amplification of *KRAS* exon 2 was performed using one of the following primers depending on the source of DNA: DNA extracted from non-fixed samples (*KRAS* Primer 1: Forward 5’ – AAG GTA CTG GTG GAG TAT TTG – 3’ and Reverse 5’ – GTA CTC ATG AAA ATG GTC AGA G – 3’, expected bp length 295), DNA extracted from FFPE samples (*KRAS* Primer 2: Forward 5’ –AAGGCCTGCTGAAAATGACTG – 3’ and Reverse 5’ – AGAATGGTCCTGCACCAGTAA – 3’, expected bp length 170; or KRAS Primer 3: Forward 5’ – ACTTGTGGTAGTTGGACCT – 3’ and Reverse 5’ – CCTCTATTGTTGGATCATATT – 3’, expected bp length 98). DNA extracted from FFPE specimens are known to be degraded and often require short length PCR amplicon to allow for a successful PCR amplification. DNA extracted from FFPE tissue not amenable to successful PCR amplification and Sanger sequencing using Primer 2 (170bp PCR fragment) were reanalyzed using Primer 3 (98bp PCR fragment). PCR reactions were carried out on a C1000 Thermal Cycler (Bio-Rad) with Platinum PCR SuperMix High Fidelity Kit (Invitrogen) using total volumes of 50 μL per reaction according to the manufacturer’s protocol. The reaction conditions were as follows: denaturation at 94°C for 30 seconds, annealing at 55°C for 30 seconds, and extension at 68°C for 45 seconds for a total of 40 cycles.

The PCR products were purified using the QIAquick PCR Purification Kit (Qiagen) and eluted into 50 uL of nuclease-free water (Qiagen). DNA was diluted to a concentration of 10 ng/uL based on Nanodrop quantification of the PCR product. Automated dideoxy terminator sequencing was performed by capillary electrophoresis by the UCLA GenoSeq Core on an ABI 3730 DNA analyzer using Big Dye Terminator chemistry (Applied Biosystems). All sequences were analyzed by manual inspection of the individual trace files using Four Peaks (Nucleobytes).

### Digital PCR

Digital PCR was performed using the QuantStudio 3D Digital PCR system (Life Technologies). Mutation analysis was performed by allele specific PCR using TaqMan probes targeting known *KRAS* mutations. Mutant and WT *KRAS* alleles are differentiated by the fluorophores attached to these probes; WT *KRAS* alleles are represented by VIC fluorescence, and mutant *KRAS* alleles are represented by FAM fluorescence. Analysis of FAM and VIC signals enables detection and quantification of mutant vs. WT allele in a sample mixture. Probes specific for G12D and G12V were used for the current analysis, as these two mutations comprise over 85% of all known *KRAS* mutations in PDAC. [13]

Reactions were performed in a 16ul reaction mixture containing gDNA (1.75ul), *KRAS* allele specific Taq-Man probe (0.85ul), QuantStudio 3D Master Mix (8.4ul) and nuclease-free water (5ul). This is then loaded onto a QuantStudio 3D Digital PCR 20K Chip (Life Technologies) using automatic chip loader (Life Technologies). The 20K chip contains 20,000 individual wells, within which DNA is randomly and uniformly distributed. The chip containing PCR reaction mixture undergoes thermocycling in the Gene Amp 9700 PCR machine. Thermocycling conditions are: 90°C for 10 minutes, followed by 60°C for 2 minutes and 98°C for 30 seconds for a total of 40 cycles, and 60°C for 2 minutes. Following the PCR reaction, the digital PCR Chip is imaged using the QuantStudio 3D Chip-Reader (Life Technologies) and the QuantStudio 3D AnalysisSuite Cloud software (Life Technologies) was used to analyze the data.

## Results

### Mutation detection sensitivity of dPCR

**Fig 1:**
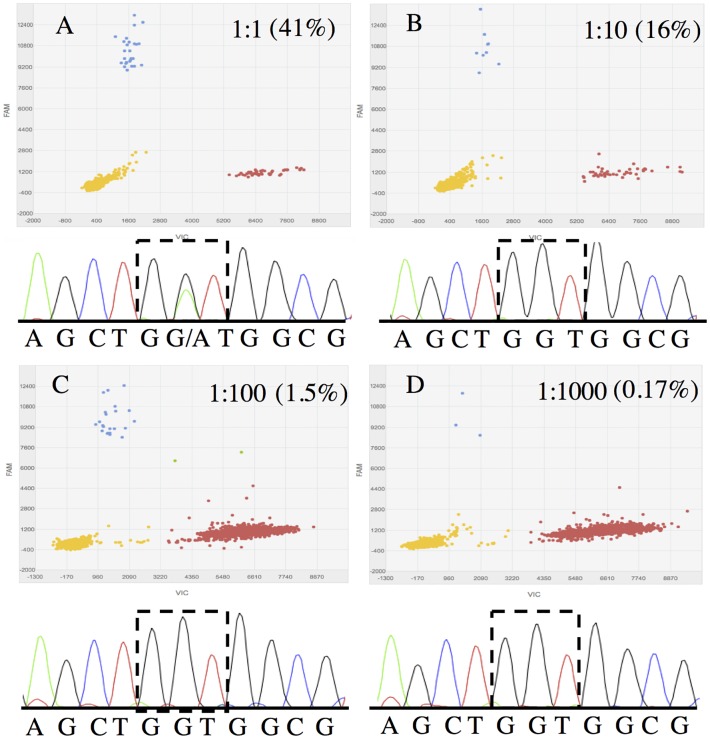
Sensitivity of mutation detection by dPCR and Sanger sequencing. DNA extracted from HPAF-II cells were serially diluted by DNA extracted from Jurkat cells to determine the limit of rare mutant detection by dPCR. Blue dots (FAM) represent presence of mutant *KRAS* (G12D) and red dots (VIC) represent WT *KRAS*. Mutant allele fractions determined by dPCR are shown in parenthesis next to dilution ratios. Same plot scales used for all four panels (A) 1:1 Mutant:WT DNA mixture; both dPCR and Sanger sequencing (GGT -> GAT) detected mutant *KRAS* allele. (B) 1:10 Mutant:WT DNA mixture; dPCR detects mutant *KRAS* allele, but Sanger sequencing shows WT *KRAS* (GGT). (C) 1: 1:100 Mutant:WT DNA mixtures: dPCR identifies mutant *KRAS* allele presence, but not in Sanger sequencing. (D) 1:1000 Mutant:WT DNA mixtures; dPCR detects *KRAS* allele, but not in Sanger sequencing.

Pancreas cancer is characterized by abundant stromal component and low neoplastic cellularity, which often results in EUS-FNA biopsy specimens containing low number of actual cancer cells compared to the large excess of normal conscripted stromal cells. Accurate mutation analysis of these samples requires a highly sensitive platform to detect rare mutant allele from the abundant WT alleles. In order to test the sensitivity of dPCR in mutation detection, we performed a serial dilution experiment using mutant *KRAS* DNA extracted from the pancreas cancer cell line HPAF-II (G12D; GGT -> GAT) and WT *KRAS* DNA extracted from the T lymphocyte cell line, Jurkat. Mixtures with mutant to WT ratio of 1:1, 1:10, 1:100 and 1:1000 were prepared and applied to dPCR and Sanger sequencing. dPCR accurately detected the presence of *KRAS* mutant allele in all mixture samples, including the one containing as little as 0.17% mutant allele fraction (1:1000 dilution) (Fig 1). Using Sanger sequencing, the presence of *KRAS* mutation was no longer detected once the mutant allele fraction reached approximately 10% (1:10 dilution), resulting in false negative results for all samples with mutant *KRAS* allele frequencies of ≤10%.

### Minimal cellular input required for dPCR mutation analysis

**Fig 2:**
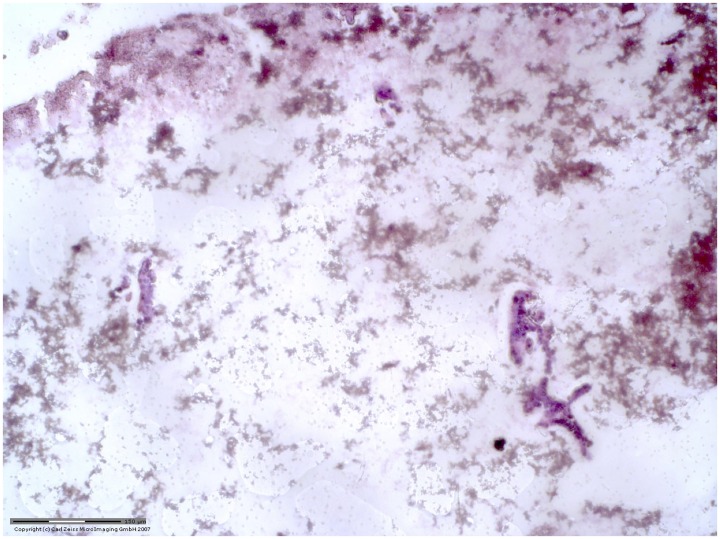
An example of FFPE pancreas EUS-FNA section containing minimal number of cells. This H&E stained 10um FFPE section was imaged with 10x magnification.

**Fig 3:**
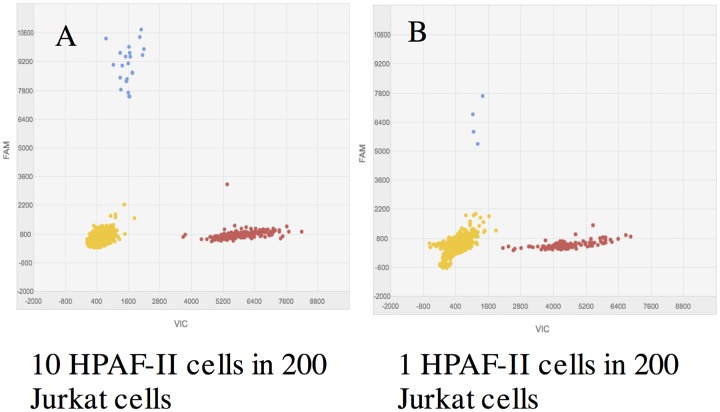
Minimal cellular input required for mutant allele detection by dPCR. HPAF-II cells and Jurkat cells were laser-microdissected to obtain exact number of cells. 1 and 10 HPAF-II cells and 200 Jurkat cells were laser-microdissected. Mixtures of 1 HPAF-II cells in 200 Jurkat cells and 10 HPAF-II cells in 200 Jurkat cells were prepared and DNA extracted. (A) dPCR was successful using DNA extracted this 210-cell mixture. Mutant *KRAS* alleles from the 10 HPAF-II cells were accurately detected. (B) dPCR detected the presence of mutant *KRAS* allele in a single HPAF-II cell in the mixture of 1 HPAF-II cell and 200 Jurkat cells.

EUS-FNA biopsy specimens often contain very limited cellular material and are frequently deemed insufficient for molecular analysis and/or cytology evaluation. An example of pauci-cellular FFPE EUS-FNA specimen is shown in Fig 2. In order to determine the minimum cellular input required for mutation analysis using dPCR, we used laser microdissection to cut and isolate a known number of cells and applied their extracted DNA to dPCR. Specifically, 1 HPAF-II cell, 10 HPAF-II cells and 200 Jurkat cells were laser microdissected. Mixture of 1 HPAF-II cell in 200 Jurkat cells and 10 HPAF-II in 200 Jurkat cells were made. DNA was then extracted from these 201-cell and 210-cell mixture samples and applied to dPCR. dPCR *KRAS* mutation detection was successful using the minimal amount of DNA extracted from approximately 200 cells (Fig 3). Furthermore, a single mutant tumor cell (HPAF-II) could be detected within an abundance of normal cells using dPCR. (Fig 3B)

### Digital PCR probe cross-reactivity

**Fig 4:**
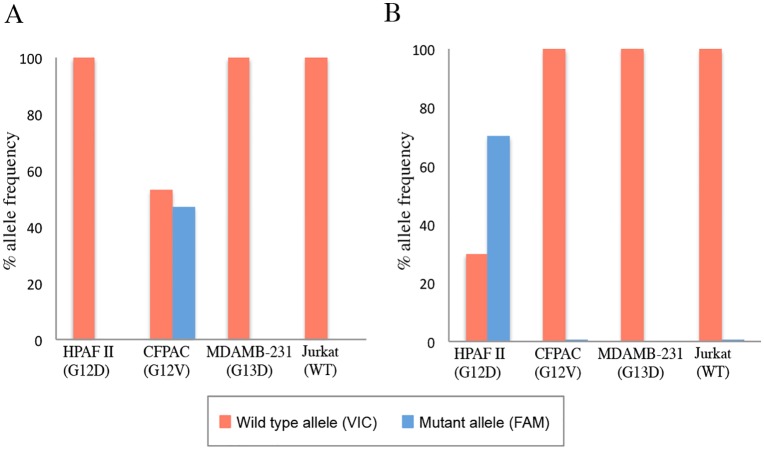
Cross-reactivity of allele-specific TaqMan probes for *KRAS* mutation subtypes. (A) DNA extracted from HPAF-II (G12D *KRAS* mutation; heterozygous with 3:1 mutant allele specific amplification), CFPAC (G12V *KRAS* mutation: heterozygous with 1:1 mutant to WT alleles), MDAMB-231 (G**13**D *KRAS* mutation) and Jurkat cells (WT *KRAS*) were analyzed by dPCR using the *KRAS*_520 probe that is specific for G12V mutant subtype. Presence of the FAM fluorescence signal, which is indicative of mutant *KRAS* allele, was seen only in the dPCR reaction that used DNA extracted from CFPAC cells. (B) The same groups of DNA were analyzed using the *KRAS*_521 probe that is specific for G12D mutation subtype. As expected, FAM signal was seen only in the dPCR reaction that used DNA extracted from HPAF-II cells.

Allele specific TaqMan probes used in dPCR were tested for non-specific detection of different *KRAS* mutant subtypes and WT *KRAS*. DNA extracted from HPAF-II (heterozygous *KRAS* G12D mutation), CFPAC (heterozygous *KRAS* G12V mutation), MDAMB-231 (heterozygous *KRAS* G**13**D mutation) and Jurkat (WT *KRAS*) cells were subjected to dPCR assays using TaqMan probes specific for either *KRAS* mutation G12D (TaqMan probe *KRAS*_521) or *KRAS* mutation G12V (TaqMan probe *KRAS*_520). As shown in Fig 4, TaqMan probes were highly specific to their respective *KRAS* mutation subtypes. When the *KRAS*_520 probe was used in dPCR *KRAS* mutation analysis, FAM-fluorescent signal indicating the presence of mutant *KRAS* was observed only in DNA extracted from CFPAC cells. (Fig 4A) Similarly, when *KRAS*_521 probe was used, FAM-fluorescent signal was observed only in DNA extracted from HPAF-II cells. (Fig 4B)

### *KRAS* mutation analysis using FFPE EUS-FNA pancreas biopsy specimens

Archived cell blocks of pancreas EUS-FNA specimens represent a vast source of tumor tissue for molecular analysis, but a large subset of these FFPE specimens are pauci-cellular and/or contain degraded DNA that are inadequate in quantity and quality for conventional molecular analysis.[3] We evaluated dPCR for its ability to perform accurate and sensitive molecular analysis using limited amount of tumor tissues available from FFPE EUS-FNA pancreas biopsy specimens. To do so, we obtained a single 10um section from cell blocks containing pancreas EUS-FNA biopsy specimens and extracted DNA from these FFPE sections. Estimated total cellularity used for dPCR assay ranged between 100–2000 cells per sample, with the majority of them containing <600 cells (Table 1). *KRAS* mutations were found in 19 of 19 patients with a preoperative cytological diagnosis of adenocarcinoma and 9 of 10 patients with atypical cells. No *KRAS* mutations were detected in the 5 patients with cytological evaluation showing no evidence of cancer.

**Table 1:** dPCR *KRAS* mutation analysis in FFPE EUS-FNA pancreas biopsy specimens.

	Cytological Diagnosis		
	Adenocarcinoma (N = 19)	Atypical cells (N = 10)	Negative for malignancy (N = 5)
**Age (years, SD)**	69 (10.8)	71 (10.4)	64 (15.6)
**Surgically resected (N, %)**	20 (100%)	10 (100%)	0
**Surgical pathology diagnosis (N)**	PDAC (19)	PDAC (1) MD-IPMN gastric subtype (5) BD-IPMN gastric subtype (3) PanIN (1)	N/A (no surgery)
**FFPE EUS-FNA block cellularity (N, %) [number of cells/10-um section]**			
**100–200**	7 (31%)	2 (22%)	0
**201–400**	5 (23%)	2 (22%)	2 (40%)
**401–600**	5 (23%)	3 (33%)	1 (20%)
**>600**	5 (23%)	2 (22%)	2 (40%)
**dPCR *KRAS* mutation analysis (N)**			
**Mutated *KRAS***	19	9	0
**WT *KRAS***^a^	0	1^b^	5

^a^dPCR indicates absence of G12D or G12V mutation

^b^ WT *KRAS* found in the patient with BD-IPMN

**Abbreviations:** dPCR; digital PCR, WT; wild-type, PDAC; pancreas adenocarcinoma, MD-IPM; main duct intraductal papillary mucinous neoplasm, BD-IPMN; branch duct intraductal papillary mucinous neoplasm, PanIN; pancreas intraepithelial neoplasia

To confirm these findings, we performed Sanger sequencing on matching surgically resected pancreas tissues. We selectively chose portions of the surgical specimen with high tumor purity to allow for confirmatory *KRAS* mutation analysis using Sanger sequencing. We found 100% concordance in *KRAS* mutation status between dPCR in limited FFPE EUS-FNA biopsy specimens and Sanger sequencing in highly cellular surgically resected tissues (Table 2).

**Table 2:** Comparison of *KRAS* mutation analysis in FFPE EUS-FNA by dPCR and surgically resected tissue by Sanger sequencing^a^.

		dPCR in EUS-FNA FFPE sections		
	*KRAS* mutation status	WT	G12V or G12D^b^ (1 mutation)	G12V and G12D (2 mutations)
**Sanger sequencing in surgically resected tissues**	WT	1	0	0
	G12V or G12D (1 mutation)	0	22	4
	G12V and G12D (2 mutations)	0	0	1

^**a**^Cytologically benign samples are excluded from this table as none of these patients underwent surgical resection

^b^*KRAS* mutation subtypes matched between dPCR and Sanger sequencing for all patients in this category

**Abbreviations:** dPCR; digital PCR, WT; wild-type

### Detection of additional *KRAS* mutation subtypes with dPCR that are not seen in sanger sequencing

Interestingly, dPCR analysis in pancreas EUS-FNA biopsy specimens identified additional *KRAS* mutation subtypes that were not seen with Sanger sequencing in matching surgically resected pancreas specimens. Presence of multiple *KRAS* mutations (G12V and G12D) was observed in 5 patients using dPCR, all of whom had FNA cytological diagnosis of “adenocarcinoma” and surgical pathologic diagnosis of PDAC. Sanger sequencing detected more than one *KRAS* mutations in only 1 of these patients, despite using matching surgical tissues that are significantly more cellular and presumably more representative of the heterogeneity of the PDAC mass compared to a limited EUS-FNA biopsy specimen (Table 2).

**Fig 5:**
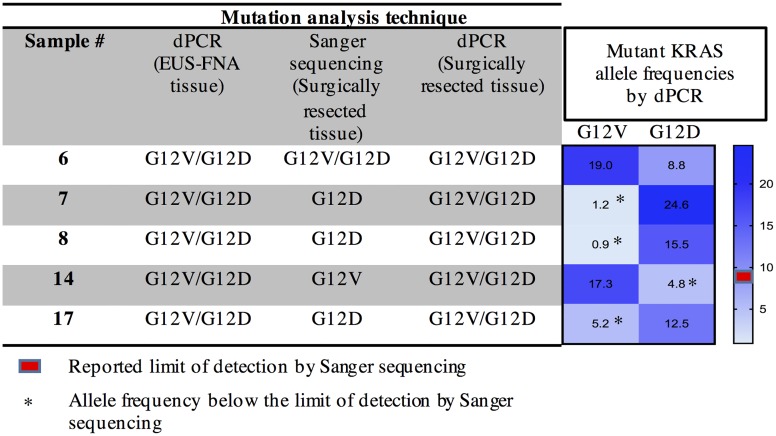
FFPE EUS-FNA specimens shown to have more than one *KRAS* mutations. dPCR *KRAS* analysis in FNA specimens were compared to Sanger sequencing done in matching surgically resected specimens. Since Sanger sequencing is known to have limited sensitivity, these surgical tissues were further analyzed by dPCR, which showed concordance with findings noted in FNA specimens. Mutant allele fractions for the mutant *KRAS* subtypes not seen in Sanger sequencing were all well below the theoretical limit of detection by Sanger sequencing.

To confirm this finding, we applied dPCR to surgically resected pancreas tissues of the same 5 patients that were found to have double *KRAS* mutations in corresponding EUS-FNA biopsies. As shown in Fig 5, we found double *KRAS* mutation subtypes in all of these 5 patients using dPCR, confirming the genotyping results observed in EUS-FNA biopsy specimens. Furthermore, for the 4 cases in which Sanger sequencing failed to show double *KRAS* mutations, one of the two mutant *KRAS* alleles existed in frequency well below the limit of detection by Sanger sequencing, resulting in failure of Sanger sequencing to detect them (Fig 5). Taken together, dPCR was able to more thoroughly identify the true mutational status of the *KRAS* gene in PDAC patients compared to Sanger sequencing, even from limited tumor tissues available from FFPE pancreas EUS-FNA biopsy specimens.

### *KRAS* mutation analysis using fresh (non-fixed) EUS-FNA specimens

**Fig 6:**
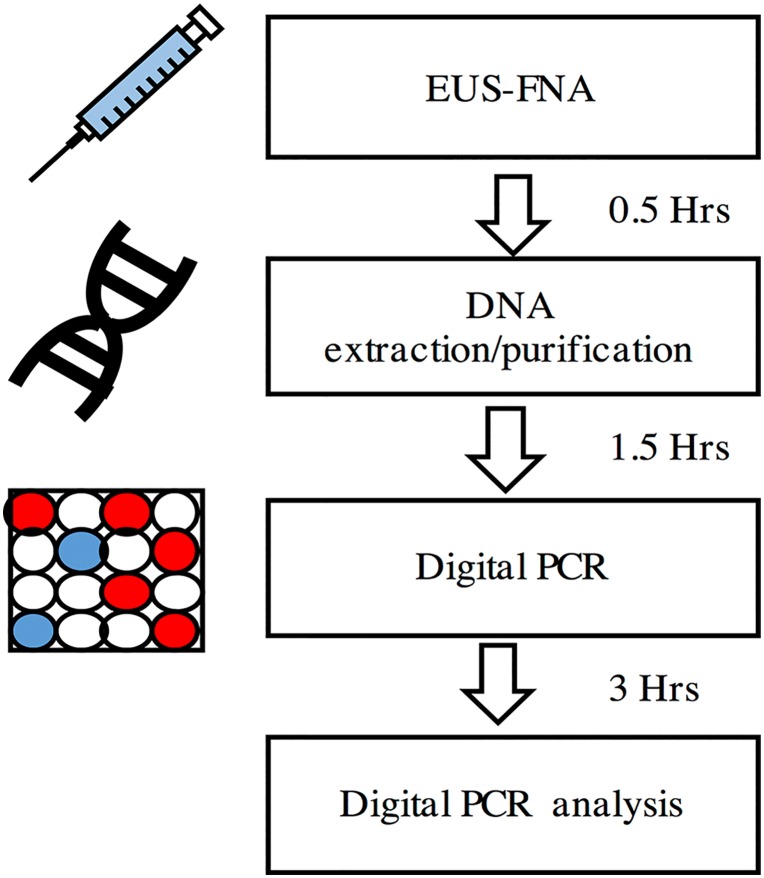
Workflow for the same-day *KRAS* mutation analysis in fresh EUS-FNA biopsy specimens using dPCR. EUS-FNA biopsy specimens are processed for DNA extraction within half an hour from the time of biopsy. Next, the extracted DNA is prepared for analysis with dPCR. The entire thermocycling protocol requires approximately 3 hours. Following completion of the PCR step, the dPCR chip is read and analyzed using the chip-reader. Output data are inspected manually for quality check and *KRAS* mutation status is determined.

Genetic information obtained from fresh EUS-FNA biopsy specimens immediately following a diagnostic endoscopic procedure allows for an enhanced point of care diagnostics and treatment guidance. We evaluated the feasibility of using dPCR as a clinical tool for *KRAS* mutation analysis in fresh EUS-FNA pancreas biopsy specimens. Clinical FNA specimens were obtained from 10 patients who underwent EUS-FNA for diagnosis of pancreas mass. Cytological and final diagnoses are as shown in Table 3. DNA extracted from these specimens were subjected to both Sanger sequencing and dPCR for *KRAS* mutation detection. Our workflow for the same-day dPCR *KRAS* mutation analysis is depicted in Fig 6. Using this workflow, we were able to determine *KRAS* mutation status within 5 hours following the EUS-FNA procedure.

**Table 3:** *KRAS* mutation analysis in fresh (non-fixed) EUS-FNA biopsy specimens.

Patient #	Mutation analysis results				Diagnosis	
	dPCR (% allele fraction)		Sanger sequencing	Sanger sequencing: LMD-WGA product	Cytological diagnosis	Final diagnosis
**35**	G12V (1.9%)	G12D (1.5%)	WT	G12V/G12D	Adenocarcinoma	PDAC
**36**	G12V (0.5%)	G12D (2.6%)	WT	G12V/G12D	Adenocarcinoma	PDAC
**37**	G12D (9.1%)		WT	G12D	Adenocarcinoma	PDAC
**38**	G12V (12.8%)		G12V	-	Adenocarcinoma	PDAC
**39**	G12V (5.9%)	G12D (10.0%)	WT	G12V/G12D	Atypical cells	Metastatic PDAC
**40**	G12V (12.8%)	G12D (7.1%)	Insufficient material	G12D	Adenocarcinoma	Metastatic PDAC
**41**	G12V (6.4%)	G12D (7.1%)	Insufficient material	G12V/G12D	Atypical cells	Chronic pancreatitis
**42**	G12D (11%)		G12D	-	Adenocarcinoma	PDAC
**43**	G12D (2.9%)		WT	G12D	Adenocarcinoma	Cholangiocarcinoma
**44**	G12D (30.3%)		G12D	-	Adenocarcinoma	PDAC

**Abbreviations:** dPCR; digital PCR, WT; wild-type, LMD; laser micro-dissection, WGA: whole genome amplification, PDAC; pancreas adenocarcinoma

We found dPCR to significantly improve the rate of *KRAS* mutation detection in pancreas EUS-FNA specimens over Sanger sequencing, the current gold standard for clinical sequencing. Using dPCR, we detected at least one subtype of *KRAS* mutation in all of the 10 EUS-FNA specimens tested (Table 3). On the other hand, Sanger sequencing only detected *KRAS* mutations in 3 of the 10 cases. All of these 3 cases had mutant *KRAS* allele frequency of >10%, high enough for Sanger sequencing to detect the *KRAS* mutation. Of the remaining 7 cases, 5 were detected as WT *KRAS* and 2 had inadequate amount of DNA for Sanger sequencing. For all of the 5 cases in which WT *KRAS* sequence was obtained, we noted a mutant allele frequency below 10% by dPCR. (Table 3) These findings validated our view that low tumor cell purity and cellular contents in pancreas EUS-FNA biopsy specimens are truly barriers to an accurate molecular analysis, and that dPCR could overcome them to provide accurate and reliable tumor genotyping.

An alternative method to overcoming the low cellularity and low tumor cell purity in some FNA specimens is to use laser micro-dissection (LMD) to isolate areas composed of mostly cancerous cells, and then amplify the extracted DNA using whole genome amplification (WGA). The resulting DNA product thus contains a high amount (>2ng) of DNA with enhanced tumor purity. The entire process is time-consuming and labor intensive and currently impractical for routine clinical use. However, this method allows for Sanger sequencing to be used and thus enables confirmation of the tumor mutation status using the current clinical gold standard. In order to further examine those samples with discrepant results between dPCR and Sanger sequencing, we utilized this alternative method (LMD-WGA) to confirm the accuracy of dPCR *KRAS* mutation analysis. To do so, we obtained FFPE sections from cell blocks prepared at the time of the same EUS-FNA procedures. Areas with high tumor cell purity were laser micro-dissected. Extracted DNA then underwent WGA to produce adequate amount of DNA for Sanger sequencing. Using this method, we detected the presence of *KRAS* mutations in the remaining 7 patients with Sanger sequencing, confirming the result observed with dPCR (Table 3, Sanger Sequencing: LMD-WGA). In one patient (patient #40, Table 3), confirmatory Sanger sequencing could not identify the G12V *KRAS* mutant allele that was present in the dPCR result. This may be explained by the fact that two different passes of FNAs were used for fresh specimen and archived cell block, resulting in different portions of the heterogeneous PDAC mass being biopsied. Additionally, allele drop out (ADO) is a frequent phenomenon observed in whole genome amplification, which may have led to the loss of heterozygosity in the *KRAS* gene in this particular sample.[20]

## Discussion

As more therapeutic decisions in cancer treatment are driven based on tumor genetic information, the ability to obtain accurate genetic information from tumor specimens becomes instrumental to the effective management of cancer patients. In clinical practice, tumor specimens are often obtained using FNAs, especially following disease progression or recurrence. Furthermore, FNA biopsies represent the only means of obtaining tumor tissues in a large subset of patients with metastatic or locally advanced diseases who will not undergo surgical resection of their primary tumor. This is especially true in PDAC patients, as >50% are diagnosed with distant diseases. In order to use pancreas FNA specimens as a source of tumor tissue for tumor genotyping, 2 major hurdles must be overcome. First is the inadequacy of DNA material extracted from a limited biopsy specimen; pauci-cellular FNA specimens do not provide adequate amount of DNA for molecular analysis. Second is the low tumor purity that complicates FNAs of many PDAC masses, which can result in overshadowing of tumor cells by the abundant normal cells.

In the current study, we evaluated the utility of dPCR as an effective strategy for mutation analysis in pancreas EUS-FNA biopsy specimens. We showed through our cell line experiments that dPCR was both highly sensitive (mutation detection sensitivity of 0.17%) and feasible with low input number of starting cells (approximately 200 cells). We further showed that dPCR accurately detected *KRAS* mutations in both formalin-fixed and fresh pancreas EUS-FNA specimens, and identified additional *KRAS* mutation subtypes that could not be detected by the current clinical gold standard method of Sanger sequencing. These features make dPCR an ideal technology for mutation analysis in limited tumor biopsy specimens of FNAs, especially from less cellular cancers such as PDAC.

One notable finding from our current study was the ability of dPCR to detect more than one *KRAS* mutation subtypes within a tumor, depicting the presence of intratumoral heterogeneity using only FNA biopsy specimens. The presence of double *KRAS* mutations (G12D and G12V) was detected in 10 of 38 (26%) pancreas FNA specimens with mutations in the *KRAS* gene. Prior studies on *KRAS* mutation detection in PDAC specimens also reported similar findings when highly sensitive mutation analysis methods were used.[9, 14, 21] In one study comparing the performance of next generation sequencing (NGS), allele specific locked nucleic acid qPCR (ASLNAqPCR) and Sanger sequencing in *KRAS* mutation detection from PDAC specimens, multiple *KRAS* mutation subtypes were found in 23%, 16% and 6.5% of specimens with mutation in the *KRAS* gene, respectively.[9] Our findings suggest that the highly sensitive nature of dPCR allows for detection of rare subclones within a tumor, signifying heterogeneity using a FNA biopsy sample.

Aside from dPCR, various other methodologies exist for sensitive mutational analysis in tumor specimens, including but not limited to pyrosequencing, allele specific locked nucleic acid PCR, COLD-PCR and next-generation sequencing (NGS).[9, 22, 23] Although these methodologies can offer sensitive mutation detection of up to 1% or less and are viable alternatives, their routine use in clinical settings may be limited by their complexity, time-consuming process, cost and/or need for relatively high amount of DNA similar to Sanger sequencing. COLD-PCR, for example, requires optimal critical temperature (Tc) to be determined for each amplicon tested.[22] Furthermore, a suitable critical temperature differentiating between WT and mutant DNA may not be available in some cases.

NGS, also known as massive parallel sequencing, represents an innovative and widely utilized sequencing technology in both research and clinical settings. Digital PCR and NGS have distinct performance characteristics that define their roles in the clinical laboratory. First, while NGS is capable of achieving a mutation detection sensitivity approaching that of dPCR (~0.1%) through deep sequencing, NGS incurs a considerably higher cost compared to dPCR. [23] Therefore, when the primary goal is to detect a known target mutation within a single gene, dPCR holds a significant economic advantage over NGS. However, the primary strength of NGS is in its ability to perform massive parallel sequencing to detect a wide array of known and unknown mutations. It outperforms dPCR in both throughput and cost when the goal of analysis is to evaluate multiple genes for potentially important aberrations. Thus, dPCR and NGS have unique strengths that make them suitable for different applications. Another important distinction between the two technologies lies in their genomic template input requirements. In our current analysis, we chose to work with limited biopsy specimens of FFPE pancreas needle aspirates in order to highlight the strength of dPCR in working with such samples. From our prior experience, we have observed that these limited samples largely fail library preparation for NGS due to their insufficient DNA quantity and quality. This is important, as it indicates that samples not amenable to NGS targeted sequencing may still be successfully interrogated with dPCR.

Given the aforementioned strengths, dPCR has potential for routine clinical use. Accurate determination of *KRAS* mutation status from pancreas EUS-FNA biopsy specimens has important clinical implications to the diagnostic, prognostic and therapeutic aspects of PDAC patient care.[15–17, 24, 25] To date, numerous studies have investigated the diagnostic role of adding *KRAS* mutation analysis to biopsy specimens.[18, 26, 27] A recent meta-analysis by Fuccio et, al. showed that the addition of *KRAS* testing in EUS-FNA specimen not only increased the sensitivity of diagnosing PDAC, but also reduced the need for a repeat EUS-FNA biopsy in inconclusive cases.[28] With the improved *KRAS* mutation detection facilitated by dPCR, *KRAS* analysis in EUS-FNA can be performed more accurately, even in FNA specimens previously thought to be inadequate for molecular analysis. In addition to diagnostics, numerous reports now suggest the role of specific *KRAS* mutation subtypes in prediction of prognosis and therapeutic responses.[15–18] Furthermore, *KRAS* mutation subtype-specific inhibitor was recently reported as a potential novel therapy.[19] These emerging reports indicate the potential for *KRAS* mutation subtyping to guide therapeutic decisions in the near future, and signify the importance of identifying all *KRAS* mutation subtypes present within a tumor.

Lastly, our findings indicated that *KRAS* mutations are not exclusive to PDAC lesions, but can also occur in other pancreatobiliary lesions as well, including chronic pancreatitis, cholangiocarcinoma and IPMNs. *KRAS* mutations are well known to exist in these non-PDAC lesions, and numerous prior repots have observed similar findings.[25, 29, 30] Although *KRAS* mutation is known to occur in some IPMNs, detection of mutant *KRAS* in 7/8 (88%) patients was higher than initially expected. However, on review of the clinical pathology reports we noted that all of these IPMN patients were of the gastric-subtype. *KRAS* mutations are common in gastric-type IPMNs and occurs in 70–80% of these lesions, which is significantly higher than the rate seen in other IPMN subtypes.[29] Interestingly, a recent report on *KRAS* mutation analysis using NGS also reported a similarly high *KRAS* mutation rate of 83.3% in patients with IPMNs, although the exact histologic subtypes of these IPMNs are unknown. [9]

In conclusion, we report the performance of dPCR for highly sensitive mutation analysis in clinical EUS-FNA pancreas specimens. Our results indicate that dPCR allows FNA samples to be used as a reliable source of tumor tissue for genetic analysis. Our findings further suggest that dPCR may be a practical alternative to Sanger sequencing, allowing detection of additional *KRAS* mutations present at allele frequencies well below ten percent. Although our current analysis was performed only in pancreas EUS-FNAs focusing on alterations in the *KRAS* gene, mutations in other cancer-related genes should be readily detectable utilizing a dPCR platform. Thus, dPCR has the potential to function as an effective molecular analysis tool and will allow for the full potential of FNA samples to be exploited in guiding precision oncology strategies.

## References

[1] Hanahan D, Weinberg Robert A. Hallmarks of Cancer: The Next Generation. Cell. 2011;144(5):646–74. 10.1016/j.cell.2011.02.013 21376230

[2] Garrawa LA, Verweij J, Ballman KV. Precision oncology: An Overview. Journal of Clinical Oncology. 2013;31(15):1803–5. Epub April 15, 2013. 10.1200/JCO.2013.49.4799 23589545

[3] Kanagal-Shamanna R, Portier BP, Singh RR, Routbort MJ, Aldape KD, Handal BA, et al Next-generation sequencing-based multi-gene mutation profiling of solid tumors using fine needle aspiration samples: promises and challenges for routine clinical diagnostics. Modern Pathology. 2013;27(2):314–27. 10.1038/modpathol.2013.122 23907151

[4] LeBlanc JK C D, Al-Assi MT M K, Imperiale T, Tao LC, Vallery S, DeWitt J, Sherman S, Collins E. Optimal number of EUS-guided fine needle passes needed to obtain a correct diagnosis. Gastrointest Endosc. 2004;59:475–81. 15044881 10.1016/s0016-5107(03)02863-3

[5] Graham JS, Jamieson NB, Rulach R, Grimmond SM, Chang DK, Biankin AV. Pancreatic cancer genomics: where can the science take us? Clinical Genetics. 2015;88(3):213–9. 10.1111/cge.12536 25388820

[6] Court CM, Ankeny JS, Hou S, Tseng H-R, Tomlinson JS. Improving pancreatic cancer diagnosis using circulating tumor cells: prospects for staging and single-cell analysis. Expert Review of Molecular Diagnostics. 2015;15(11):1491–504. 10.1586/14737159.2015.1091311 26390158 PMC4893319

[7] Perincheri S, Hui P. KRAS mutation testing in clinical practice. Expert Review of Molecular Diagnostics. 2014;15(3):375–84. 10.1586/14737159.2015.986102 25487540

[8] Gonzalez de Castro D, Angulo B, Gomez B, Mair D, Martinez R, Suarez-Gauthier A, et al A comparison of three methods for detecting KRAS mutations in formalin-fixed colorectal cancer specimens. Br J Cancer. 2012;107(2):345–51. 10.1038/bjc.2012.259 22713664 PMC3394984

[9] de Biase D, Visani M, Baccarini P, Polifemo AM, Maimone A, Fornelli A, et al Next generation sequencing improves the accuracy of KRAS mutation analysis in endoscopic ultrasound fine needle aspiration pancreatic lesions. PLoS One. 2014;9(2):e87651 10.1371/journal.pone.0087651 24504548 PMC3913642

[10] Huggett JF, Cowen S, Foy CA. Considerations for Digital PCR as an Accurate Molecular Diagnostic Tool. Clinical Chemistry. 2014;61(1):79–88. 10.1373/clinchem.2014.221366 25338683

[11] Bidshahri R, Attali D, Fakhfakh K, McNeil K, Karsan A, Won JR, et al Quantitative Detection and Resolution of BRAF V600 Status in Colorectal Cancer Using Droplet Digital PCR and a Novel Wild-Type Negative Assay. The Journal of Molecular Diagnostics. 2016;18(2):190–204. 10.1016/j.jmoldx.2015.09.003 26762843

[12] Lamy P-J, Castan F, Lozano N, Montélion C, Audran P, Bibeau F, et al Next-Generation Genotyping by Digital PCR to Detect and Quantify the BRAF V600E Mutation in Melanoma Biopsies. The Journal of Molecular Diagnostics. 2015;17(4):366–73. 10.1016/j.jmoldx.2015.02.004 25952101

[13] Bryant KL, Mancias JD, Kimmelman AC, Der CJ. KRAS: feeding pancreatic cancer proliferation. Trends in Biochemical Sciences. 2014;39(2):91–100. 10.1016/j.tibs.2013.12.004 24388967 PMC3955735

[14] Kinugasa H, Nouso K, Miyahara K, Morimoto Y, Dohi C, Tsutsumi K, et al Detection ofK-rasgene mutation by liquid biopsy in patients with pancreatic cancer. Cancer. 2015;121(13):2271–80. 10.1002/cncr.29364 25823825

[15] Ogura T, Yamao K, Hara K, Mizuno N, Hijioka S, Imaoka H, et al Prognostic value of K-ras mutation status and subtypes in endoscopic ultrasound-guided fine-needle aspiration specimens from patients with unresectable pancreatic cancer. Journal of Gastroenterology. 2012;48(5):640–6. 10.1007/s00535-012-0664-2 22983505

[16] Hamidi H, Lu M, Chau K, Anderson L, Fejzo M, Ginther C, et al KRAS mutational subtype and copy number predict in vitro response of human pancreatic cancer cell lines to MEK inhibition. British Journal of Cancer. 2014;111(9):1788–801. 10.1038/bjc.2014.475 25167228 PMC4453732

[17] Bournet B, Muscari F, Buscail C, Assenat E, Barthet M, Hammel P, et al KRAS G12D Mutation Subtype Is A Prognostic Factor for Advanced Pancreatic Adenocarcinoma. Clinical and Translational Gastroenterology. 2016;7(3):e157. 27010960 10.1038/ctg.2016.18 PMC4822095

[18] Bournet Barbara, Buscail Camille, Muscari Fabrice, Cordelier Pierre, Buscai L. Targeting KRAS for diagnosis, prognosis, and treatment of pancreatic cancer: Hopes and realities. European Journal of Cancer. 2016;54:75–83. 10.1016/j.ejca.2015.11.012 26735353

[19] Lito P, Solomon M, Li L-S, Hansen R, Rosen N. Allele-specific inhibitors inactivate mutant KRAS G12C by a trapping mechanism. Science. 2016;351(6273):604–8. 10.1126/science.aad6204 26841430 PMC4955282

[20] Court CM, Ankeny JS, Sho S, Hou S, Li Q, Hsieh C, et al Reality of Single Circulating Tumor Cell Sequencing for Molecular Diagnostics in Pancreatic Cancer. The Journal of Molecular Diagnostics. 2016;In press. 10.1016/j.jmoldx.2016.03.006 PMC5397706 27375074

[21] Azuara D, Ginesta MM, Gausachs M, Rodriguez-Moranta F, Fabregat J, Busquets J, et al Nanofluidic digital PCR for KRAS mutation detection and quantification in gastrointestinal cancer. Clinical chemistry. 2012;58(9):1332–41. 10.1373/clinchem.2012.186577 22745110

[22] Li J, Wang L, Mamon H, Kulke MH, Berbeco R, Makrigiorgos GM. Replacing PCR with COLD-PCR enriches variant DNA sequences and redefines the sensitivity of genetic testing. Nature Medicine. 2008;14(5):579–84. 10.1038/nm1708 18408729

[23] Valero V 3rd, Saunders TJ, He J, Weiss MJ, Cameron JL, Dholakia A, et al Reliable Detection of Somatic Mutations in Fine Needle Aspirates of Pancreatic Cancer With Next-generation Sequencing: Implications for Surgical Management. Ann Surg. 2016;263(1):153–61. 10.1097/SLA.0000000000001156 26020105 PMC4662640

[24] Takahashi K, Yamao K, Okubo K, Sawaki A, Mizuno N, Ashida R, et al Differential diagnosis of pancreatic cancer and focal pancreatitis by using EUS-guided FNA. Gastrointestinal Endoscopy. 2005;61(1):76–9. 15672060 10.1016/s0016-5107(04)02224-2

[25] Ogura T, Yamao K, Sawaki A, Mizuno N, Hara K, Hijioka S, et al Clinical impact of K-ras mutation analysis in EUS-guided FNA specimens from pancreatic masses. Gastrointestinal Endoscopy. 2012;75(4):769–74. 10.1016/j.gie.2011.11.012 22284089

[26] Bournet B. Role of endoscopic ultrasound in the molecular diagnosis of pancreatic cancer. World Journal of Gastroenterology. 2014;20(31):10758 10.3748/wjg.v20.i31.10758 25152579 PMC4138456

[27] Ankeny JS, Court CM, Hou S, Li Q, Song M, Wu D, et al Circulating tumour cells as a biomarker for diagnosis and staging in pancreatic cancer. Br J Cancer. 2016;114(12):1367–75. 10.1038/bjc.2016.121 27300108 PMC4984454

[28] Fuccio L, Hassan C, Laterza L, Correale L, Pagano N, Bocus P, et al The role of K-ras gene mutation analysis in EUS-guided FNA cytology specimens for the differential diagnosis of pancreatic solid masses: a meta-analysis of prospective studies. Gastrointestinal Endoscopy. 2013;78(4):596–608. 10.1016/j.gie.2013.04.162 23660563

[29] Nikiforova MN, Khalid A, Fasanella KE, McGrath KM, Brand RE, Chennat JS, et al Integration of KRAS testing in the diagnosis of pancreatic cystic lesions: a clinical experience of 618 pancreatic cysts. Modern Pathology. 2013;26(11):1478–87. 10.1038/modpathol.2013.91 23743931

[30] Arvanitakis M, Van Laethem JL, Parma J, De Maertelaer V, Delhaye M, Devière J. Predictive Factors for Pancreatic Cancer in Patients with Chronic Pancreatitis in Association with K-rasGene Mutation. Endoscopy. 2004;36(6):535–42. 10.1055/s-2004-814401 15202051

